# How the teacher development ecosystem influences career success: the chain mediation role of school climate and job crafting

**DOI:** 10.3389/fpsyg.2025.1596058

**Published:** 2025-09-24

**Authors:** You Xu, Biru Chang, Qiuxia Guo

**Affiliations:** ^1^School of Education Science, Nanjing Normal University, Nanjing, Jiangsu, China; ^2^School of Teacher Education, Shanwei Institute of Technology, Shanwei, Guangdong, China

**Keywords:** teacher development ecosystem, school climate, job crafting, career success, chain mediation

## Abstract

**Background:**

Teachers' career success is increasingly recognized as a multidimensional outcome shaped by individual and contextual factors. Drawing on ecological systems theory and conservation of resources theory, this study investigated how the teacher development ecosystem influences teachers' career success, focusing on the mediating roles of school climate and job crafting.

**Methods:**

The stratified random sampling was used to survey one thousand seven hundred and fifty five teachers across four provinces in China with self-reporting. All participants completed the Teacher Development Ecosystem Questionnaire, the School Climate Questionnaire, the Job Crafting Questionnaire, and the Teacher Career Success Questionnaire. Structural equation modeling was applied to examine the hypothesized relationships and mediating effects.

**Results:**

The results demonstrated that the teacher development ecosystem significantly and positively predicted teachers' career success. School climate partially mediated this relationship, and job crafting also acted as a mediator. Moreover, school climate and job crafting jointly exerted a chain mediation effect, indicating that a positive school climate promotes teachers' job crafting behaviors, which subsequently enhance their career success. Notably, the findings suggested that job crafting is necessarily mediated by school climate within the chained mediation model.

**Conclusion:**

This study extended the application of ecological systems theory to teachers' career development by uncovering the mechanism through which the teacher development ecosystem contributes to career success. The results provide practical implications for fostering supportive school climates and encouraging job crafting behaviors as strategies to enhance teachers' career outcomes.

The career success of teachers has profound implications for both the educational system and society (Li et al., 2023). Specifically, teachers with high job satisfaction are more effective in enhancing children's cognitive development and promoting academic progress. They are also more likely to demonstrate higher levels of professional engagement (Melesse and Belay, 2022). Previous research has established a link between the ecology of teacher development and career success, with school climate serving as the socio-psychological context of teachers' work (Johnson et al., 2007) and providing essential resources for professional growth. Teachers with access to abundant external resources tend to achieve higher levels of professional development, and social support positively influences career success (Yuh and Choi, 2017). Job crafting plays a proactive strategic role in this process. Although existing studies highlight the significance of teacher development ecosystem, school climate, and job crafting in career success, the literature lacks a comprehensive explanation of the relationships among these variables.

## Teacher development ecosystem and teacher career success

Career success is defined as positive psychological or work-related outcomes or achievements that accumulate as a result of one's work experience (Seibert et al., 1999). Career success arises from the dynamic interplay between external environmental factors and employees' proactive agency (Yu et al., 2022). In other words, teachers' career success is influenced by teacher development ecosystem. Teacher development ecosystem is a multidimensional system, which is rooted in Bronfenbrenner (1979) ecological systems theory and expanded by Zastrow and Hessenauer (2004) social-ecological theory.

The teacher development ecosystem emphasizes the dynamic interaction between teachers and their environments. It positions teachers within a network of influences that shape their professional experiences. Chong and Lu (2021) highlighted that teachers' career success was influenced by factors such as educational policies, social environment, and individual characteristics of education practitioners. Existing research explained that teacher development ecosystem influenced teachers' career success through the availability or scarcity of resources (Diab and Green, 2024). Based on this, we hypothesized:

***Hypothesis 1*: Teacher development ecosystem might positively predict teachers' career success**.

## School climate as a mediating variable

Certain contextual factors, such as teacher collaboration, the quality of teacher–student relationships, and job autonomy (Weiland, 2021), can be collectively categorized under the broad concept of school climate. Prior studies indicate that external environmental factors shape school climate, which subsequently impacts teachers' professional engagement (Martinsone and ŽydŽiunaite, 2023). Gumuseli and Eryilmaz (2011) found that policy-driven accountability measures could exacerbate tensions between teachers, colleagues, and school leaders, whereas welfare policies could encourage collaboration among colleagues and foster a positive school climate. A positive school climate, as a key resource, reduces the threat of resource loss and provides the psychological safety and support necessary for teachers to engage in the proactive, potentially risky behaviors associated with job crafting. Leithwood et al. (2008) showed that the school climate was related to teachers' professional competitiveness. Informal mentoring within schools could help new teachers rapidly acquire essential skills and strategies for professional development (Chen et al., 2024). Based on these findings, we posited:

***Hypothesis 2*: School climate might mediate the effects of teacher development ecosystem on career success**.

## Job crafting as a mediating variable

Job crafting refers to the physical and cognitive changes individuals make within the boundaries of their tasks and relationships (Wrzesniewski and Dutton, 2001). The Conservation Of Resources (COR) theory suggests that employees are motivated to acquire, protect, and build resources based on their environmental conditions and individual characteristics to cope with stress and enhance their wellbeing. Within this framework, job crafting represents a proactive strategy through which teachers align resources and cope with work demands based on their strengths, values, and career goals (Wang et al., 2020). Le Fevre and Robinson (2015) demonstrated that policies supporting teacher autonomy enable educators to adapt tasks to better align with students' needs, thereby enhancing task facilitation and cognitive crafting. Employees are more inclined to engage in job crafting to obtain additional resources when high-intensity job demands are embedded within a supportive development ecosystem (Tian et al., 2021). Based on this, we conjectured:

***Hypothesis 3*: Job crafting might mediate the effects of teacher development ecosystem on career success**.

## The chain mediation effect of school climate and job crafting

Within the teacher development ecosystem context, COR theory posits that a positive school climate serves as an initial resource that enables teachers to engage in job crafting. Within a supportive school climate, job crafting functions as a resource conservation and augmentation strategy that enables teachers to mitigate resource depletion, generate new resources, and ultimately enhance professional outcomes and career development (Hatice Güçlü Nergiz, 2024). McClelland et al. (2014) highlighted that managers should pay attention to the organizational climate and foster an organizational environment that positively influences job crafting behaviors. Similarly, Mäkikangas et al. (2017) examined the role of the organizational climate in team job crafting, finding that a resource-rich organizational environment facilitates daily team job crafting behaviors. Teachers' engagement in job crafting is facilitated by supportive collaboration with colleagues, principal support, and a positive school climate, which together provide the necessary conditions that foster both confidence and capability (Zheng et al., 2023).

**Figure 1:**
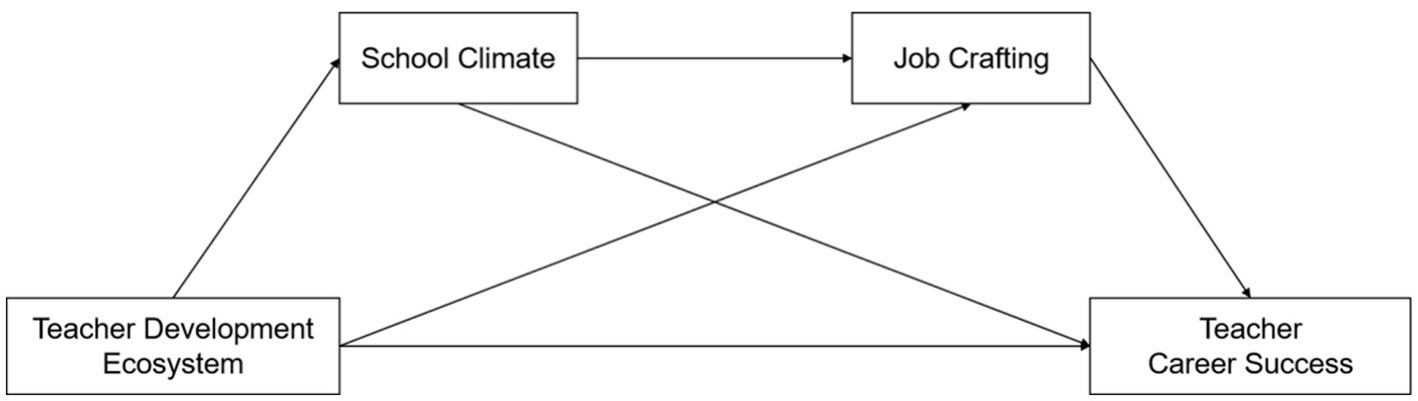
Hypothetical mediation model.

However, as previous research indicated, the teacher development ecosystem did not directly influence individual career success. Although some studies have attempted to examine the predictive relationships between school climate and career success or between job crafting and career success, they often overlook the systemic nature of the teacher development ecosystem without establishing a chain mediation model. To address this gap, we proposed the following hypothesis for empirical testing:

***Hypothesis 4*: School climate and job crafting might have a chain mediation effect** *(**Figure 1**)*.

## Methods

### Participants

The data for this study were obtained from a large-scale survey of teacher development across four Chinese provinces. Stratified sampling was employed to establish the final sample. Specifically, one province was selected from each of China's four major economic regions, and within each province, two counties were randomly chosen. The sampling ratio in each county was determined according to the number of teachers, ensuring representativeness across regions. An online questionnaire was distributed to one thousand eight hundred and eighty one teachers through www.wjx.cn (a Chinese online survey platform accessed on March 10, 2024).

A total of one thousand seven hundred and fifty five valid responses were collected. Participants ranged in age from 20 to 61 years, 1,067 teachers (60.79%) had less than 10 years of teaching experience. The study objectives and procedures were reviewed and approved by the Academic Ethics Committee of Nanjing Normal University (NNU202401002). All questionnaires were distributed anonymously via the Internet to each subject, who provided confidential information on a voluntary basis after understanding the purpose of the survey and related procedures. Participants provided informed consent after being briefed on the study's purpose and procedures. The informed consent form detailed the study's purpose, assured confidentiality, and explicitly stated that participation was voluntary and could be terminated at any time.

### Measures

#### Teacher development ecosystem questionnaire

This study employed a validated questionnaire on teacher development ecosystem in China. The scale consists of four dimensions with twenty eight items. A five-point Likert scale was used, ranging from 1 (strongly disagree) to 5 (strongly agree), with higher scores indicating a more supportive teacher development ecology. The questionnaire demonstrated strong reliability and validity. Confirmatory Factor Analysis (CFA) was conducted, yielding a Cronbach's alpha of 0.974 and fit indices of χ^2^*/df* = 4.402, GFI = 0.881, TLI = 0.908, CFI = 0.913, SRMR = 0.065, RMSEA = 0.083 [0.081, 0.085]. These results indicate that the questionnaire met the expected standards for reliability and validity.

#### School climate questionnaire

We employed the School Climate Questionnaire—Chinese Form, revised by Li et al. (2017) from the original version (Hoy and Clover, 1986). The questionnaire consists of six dimensions: principal support, principal supervision, principal restrictions, teacher engagement, teacher intimacy, and teacher disengagement, with a total of 33 items. Responses were measured on a five-point Likert scale ranging from 1 (strongly disagree) to 5 (strongly agree). CFA indicated a good model fit, with the following indices: χ^2^*/df* = 3.158, *p* < 0.001, RMSEA = 0.076, GFI = 0.84, TLI = 0.918 and CFI = 0.927. Cronbach's alpha of 0.883.

#### Job crafting questionnaire

This study employed the Job Crafting Questionnaire (JCQ) developed by Chinese scholars Qi et al. (2016) to measure teachers' job crafting levels. Based on Slemp and Vella-Brodrick (2013), this questionnaire includes five dimensions with the addition of role and skill crafting, which aligns with the realities of Chinese teachers' work environments. The questionnaire demonstrated good psychometric properties, with fit indices of χ^2^*/df* = 3.830, GFI = 0.905, TLI = 0.949, CFI = 0.902, RMSEA = 0.076 and the overall Cronbach's alpha coefficient for the scale was 0.972. Responses were rated on a five-point Likert scale ranging from 1 (strongly disagree) to 5 (strongly agree), with higher scores indicating higher job crafting levels.

#### Teacher career success questionnaire

This study used the Career Success Questionnaire (CSQ) developed by Eby et al. (2003) to assess teachers' career success. The questionnaire comprised three dimensions: intra-organizational competitiveness, inter-organizational competitiveness, and job satisfaction, with a total of 11 items. Responses were measured on a five-point Likert scale ranging from 1 (strongly disagree) to 5 (strongly agree), with higher scores indicating greater career success. In this study, CFA was conducted, yielding the following fit indices: χ^2^*/df* = 3.552, GFI = 0.846, TLI = 0.894, CFI = 0.921, SRMR = 0.057, RMSEA = 0.072. And Cronbach's alpha of 0.941, demonstrating that the questionnaire remains a valid and reliable tool for assessing career success among teachers.

### Data analysis

This study utilized SPSS (version 25.0) for data collection and analysis. Amos was used to test the mediating roles of school climate and job crafting in the relationship between the teacher development ecosystem and teachers' career success.

## Results

As all key variables in this study (*teacher development ecosystem, school climate, job crafting*, and *career success*) were self-reported by the participants, Harman's single-factor test was used to assess the potential common method bias (Podsakoff et al., 2003). Exploratory factor analysis (EFA) was conducted on the collected survey data, extracting 18 factors with eigenvalues greater than 1, which collectively explained 81.65% of the total variance. The variance explained by the largest single factor was 34.832%, which is below the critical threshold of 40%.

A bivariate correlation analysis was conducted to examine the relationships between teacher development ecosystem, school climate, job crafting, and career success. Table 1 presents the means, standard deviations, and correlation matrices of each variable. The four sets of variables exhibit significant pairwise correlations.

**Table 1:** Descriptive statistics and correlation analysis.

**Item**	**1**	**2**	**3**	**4**
1. TDE	1.00			
2. SC	0.609^**^	1.00		
3. JC	0.714^**^	0.622^**^	1.00	
4. CS	0.581^**^	0.484^**^	0.691^**^	1.00
M	4.51	4.08	4.61	4.14
SD	0.68	0.48	0.61	0.84

^**^p < 0.01 TDE, Teacher Development Ecosystem; SC, School Climate; JC, Job Crafting; CS, Career Success.

A bias-corrected bootstrapping method was used to test the mediating effects of school climate and job crafting on the relationship between teacher development ecosystem and career success. The regression analysis results indicated that, in the absence of mediating variables, teacher development ecosystem significantly and positively predicted career success (β = 0.58, *p* < 0.001), supporting H1. Additionally, teacher development ecosystem had a strong and significant predictive effect on school climate (β = 0.867, *p* < 0.001), and school climate significantly predicted job crafting (β = 0.718, *p* < 0.001). School climate significantly predicted career success (β = 0.207, *p* < 0.001). However, when including the mediating variables, the direct effect of teacher development ecosystem on career success among teachers was not significant (β = 0.076, *p* = 0.094 > 0.05), and the effect of teacher development ecosystem on job crafting was not significant (β = 0.137, *p* = 0.062 > 0.05).

For the simple mediation model of *teacher development ecosystem*→*school climate*→*career success*, the total effect was 0.751 (95% CI = [0.611, 0.827]), and the indirect effect was 0.268 (95% CI = [0.169, 0.363]), supporting H2. For the simple mediation model of *teacher development ecosystem*→*job crafting*→*career success*, the total effect was 0.649 (95% CI = [0.575, 0.713]), and the indirect effect was 0.460 (95% CI = [0.391, 0.546]), supporting H3.

**Figure 2:**
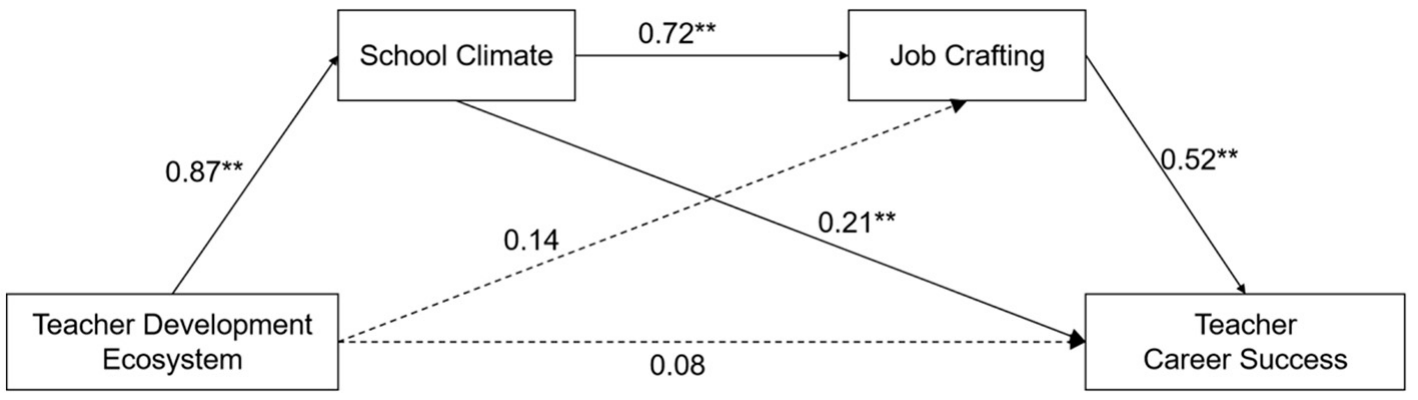
Mediation model examining the indirect relationship between teacher development ecosystem and teacher career success through school climate and job crafting. **p < 0.01.

For the chain mediation model of *teacher development ecosystem*→*school climate*→*job crafting*→*career success*, the total indirect effect was 0.573 (95% CI = [0.454, 0.685]), accounting for 83.6% of the total effect (0.649). The indirect mediation effect of teacher development ecosystem → job crafting → career success was not significant, with an effect value of 0.071 (95% CI = [-0.011, 0.150]). The mediation effect of teacher development ecosystem → school climate → job crafting → career success was significant, with an effect value of 0.322 (95% CI = [0.232, 0.433]), accounting for 49.61% of the total effect, supporting H4 (Table 2). These results indicated that the pathway between school climate and job crafting played a critical role in linking teacher development ecosystem to career success (Figure 2).

**Table 2:** Standardized total, direct, and indirect effects in the chained mediation model, along with their 95% confidence intervals.

**Pathway**	**Estimate**	**Ratio**	**95% Confidence interval**	
			**Lower**	**Upper**
1. TDE → SC → CS	0.180	27.73%	0.045	0.302
2. TDE → JC → CS	0.071	10.94%	−0.011	0.150
3. TDE → SC → JC → CS	0.322	49.61%	0.232	0.433
Total indirect effect	0.573	88.29%	0.454	0.685
Direct effect	0.076	11.71%	−0.041	0.237
Total effect	0.649	100%	0.573	0.711

TDE, Teacher Development Ecosystem; SC, School Climate; JC, Job Crafting; CS, Career Success.

## Discussion

In the chain mediation model, teacher development ecosystem did not directly predict job crafting or career success. Instead, teacher development ecosystem influenced school climate, which subsequently affected job crafting and ultimately contributed to career success. This highlights the critical role of a supportive school climate in fostering job crafting behaviors and promoting teachers' career success. These findings extended the application of the ecological systems theory in the field of teacher career development, broadening its theoretical boundaries.

First, optimizing teacher development ecosystem is a key initiative for supporting teachers in achieving career success, with school climate as a critical environmental factor that warrants particular attention. Previous research has shown that a high-quality teacher development ecosystem enhances teachers' sense of career achievement and teaching effectiveness through continuous professional development opportunities and psychological support (Fathur et al., 2024). And indicated that collaboration between schools, communities, and policies was crucial for shaping teacher career success (Vangrieken et al., 2017). This study further validated these findings and extended the applicability of ecological systems theory in educational contexts.

Second, this study found that school climate significantly mediated the relationship between teacher development ecosystem and career success, deepening the existing research on school climate (Law et al., 2024). As the most immediate and enduring contextual variable in teachers' daily work, school climate shapes teachers' psychological states and work attitudes, which in turn influence job crafting behaviors and ultimately affect career success.

Third, these findings contribute to the theoretical framework of job crafting (Wrzesniewski and Dutton, 2001). This result aligned with Bakker et al. (2007) and their Job Demands-Resources (JD-R) Model. These findings further elucidate the intrinsic connection between resource availability and job crafting (Likitha et al., 2024), showing that a positive school climate is positively related to teachers' engagement in job crafting. It also underscored the fact that job crafting was not an isolated behavior and emphasized the need for institutional support to maximize benefits.

Fourth, a positive school climate stimulates teachers' interest in and willingness to engage in job crafting, enabling them to utilize the resources provided by development ecology more effectively (Geijsel et al., 2007). This chain mediation mechanism highlighted the critical role of resource integration: a positive school climate amplified the potential effectiveness of available resources (Shao et al., 2025), whereas job crafting enabled teachers to effectively transform these resources into professional achievement (Hargreaves and Fullan, 2012). Most importantly, in the chained mediation model, teacher job crafting must be mediated by school climate. School climate significantly influenced the level of teacher job crafting, serving as a crucial intermediary mechanism in this process. This process may reflect the collectivist organizational management system in China (Zhang et al., 2022), whereby strong organizational support facilitates reciprocal individual contributions, thereby enhancing educational quality and fostering the sustainable development of teachers' careers within the broader ecosystem. Consequently, the teacher development ecosystem necessarily shapes career success through the mediating role of collective school organizational processes, rather than through direct effects at the individual level.

Although this study integrates multiple factors within the teacher development ecosystem, enriching and optimizing previous research, it employed a cross-sectional quantitative research design that limited the ability to establish causal relationships among the variables. Future research could adopt longitudinal data collection methods to provide a more in-depth understanding of how these variables interact over time and capture the dynamic processes within teacher career development. Moreover, this study relied solely on self-reported measures from the participants, which may introduce potential biases. Future research should incorporate qualitative research designs, such as interviews, to provide a more comprehensive understanding of the findings.

## Data Availability

The raw data supporting the conclusions of this article will be made available by the authors, without undue reservation. The data that support the findings of this study are available on request from You Xu (200601015@nnu.edu.cn).
